# Exosomal ncRNAs: The pivotal players in diabetic wound healing

**DOI:** 10.3389/fimmu.2022.1005307

**Published:** 2022-11-07

**Authors:** Jiuheng Shen, Xian Zhao, Youxiu Zhong, Peng Yang, Peifen Gao, Xue Wu, Xudong Wang, Wenlin An

**Affiliations:** National Vaccine and Serum Institute (NVSI), China National Biotech Group (CNBG), Beijing, China

**Keywords:** exosomes, wound healing, non-coding RNA, diabetes mellitus, delivery

## Abstract

Diabetes is the most prevalent metabolic disease in the world today. In addition to elevated blood glucose, it also causes serious complications, which has a significant effect on the quality of life of patients. Diabetic trauma is one of complications as a result of the interaction of diabetic neuropathy, peripheral vascular disease, infection, trauma, and other factors. Diabetic trauma usually leads to poor healing of the trauma and even to severe foot ulcers, wound gangrene, and even amputation, causing serious psychological, physical, and financial burdens to diabetic patients. Non-coding RNAs (ncRNAs) carried by exosomes have been demonstrated to be relevant to the development and treatment of diabetes and its complications. Exosomes act as vehicle, which contain nucleic acids such as mRNA and microRNA (miRNA), and play a role in the intercellular communication and the exchange of substances between cells. Because exosomes are derived from cells, there are several advantages over synthetic nanoparticle including good biocompatibility and low immunogenicity. Exosomal ncRNAs could serve as markers for the clinical diagnosis of diabetes and could also be employed to accelerate diabetic wound healing *via* the regulation of the immune response and modulation of cell function. ncRNAs in exosomes can be employed to promote diabetic wound healing by regulating inflammation and accelerating re-vascularization, re-epithelialization, and extracellular matrix remodeling. Herein, exosomes in terms of ncRNA (miRNA, lncRNA, and circRNA) to accelerate diabetic wounds healing were summarized, and we discussed the challenge of the loading strategy of ncRNA into exosomes.

## Introduction

Diabetes mellitus (DM) is a class of metabolic disease with defective insulin secretion or when the body cannot utilize the insulin effectively, which is caused by two factors including both genetic and environmental factors. In addition to increasing blood glucose, various complications induced by diabetes include diabetic cardiomyopathy, diabetic nephropathy, diabetic cerebrovascular complications, and diabetic trauma malunion, which create a large burden to patients. Among these complications, wound healing is impaired due to impaired angiogenesis, neuropathy, unusual inflammatory response, and dysfunction of fibroblasts (1). Thus, there is an urgent need to develop a novel treatment for wound healing by regulating this complex pathophysiology.

**Figure 1:**
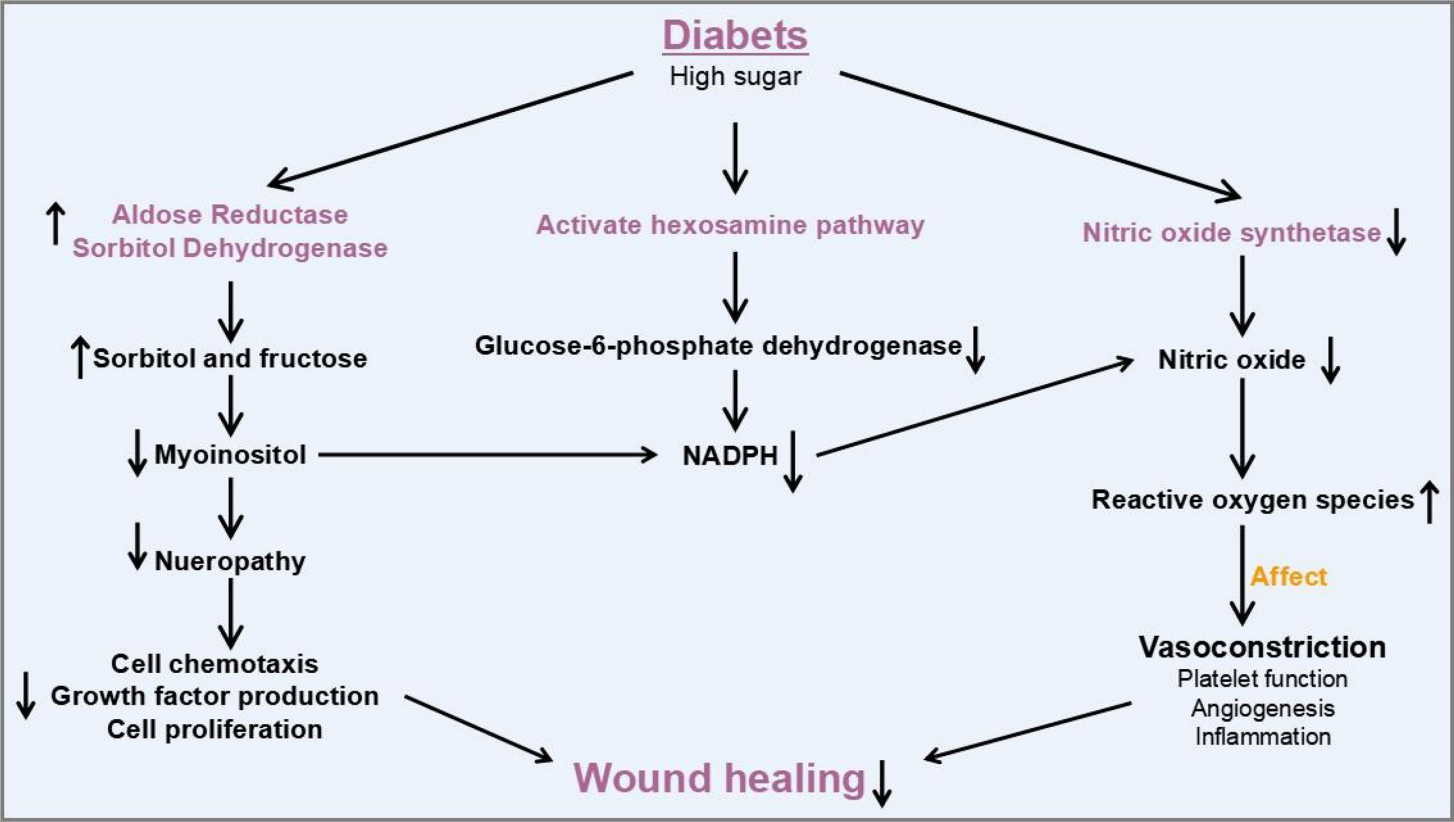
Molecular mechanisms and therapeutic potential of exosomal ncRNAs involved in diabetic wound healing.

There are several stages of wound healing such as hemostasis, inflammation, proliferation, contraction, and remodeling (2). The normal wound healing cascade is dependent on the coordination and synchronization of growth factors, matrix metalloproteinases (MMPs), cytokines, inflammatory cells, keratinocytes, fibroblasts, and endothelial cells. However, the healing process in diabetic wounds usually does not strictly follow the normal wound healing process described above and can be stalled at inflammation stage and cannot proceed to the next stage. In the diabetic state, absolute or relative insulin deficiency leads to an increase in the amount glucose and the interruption of lipid metabolism. In addition, abnormalities in energy metabolism could lead to abnormalities in immune cells and signal transduction, which play significant roles in chronic long-term inflammation of the wound and consequently in healing (**Figure 1**). A series of mechanisms such as polyol pathway, hexosamine pathway, protein kinase C (PKC) pathway, and nitric oxidase synthase pathway that lead to neuropathy impede diabetic wound healing. As a result, elevated levels of oxidative stress in diabetic patients are a major factor in impaired diabetic wound healing (3) Furthermore, diabetic wounds also suffer from hypoxia, abnormal vascularization, neurological damage, reduced number of epidermal nerves (4), reduction in the secretion of growth factors, and imbalance between accumulation of extracellular matrix components and matrix metalloproteinase remodeling. In all, these factors contributed to the delay in wound healing in diabetic patients through keeping the wound inflamed for a long period (3).

Therefore, the promotion of wound healing is an effective strategy to significantly reduce amputations and deaths in diabetic patients. In recent years, surgery was considered as an efficient approach in the treatment of diabetic foot ulcer (DFU). Stem cells and related derivative biologics have been employed as alternative for diabetic wound healing. Nevertheless, there are many drawbacks in the use of stem cells such as the risk of rejection and carcinogenesis. Over the past decades, exosomes that are secreted by cells have received much attention as a cell-free alternative owing its possessing similar functions with cells without causing rejection and carcinogenesis (5).

**Scheme 1:**
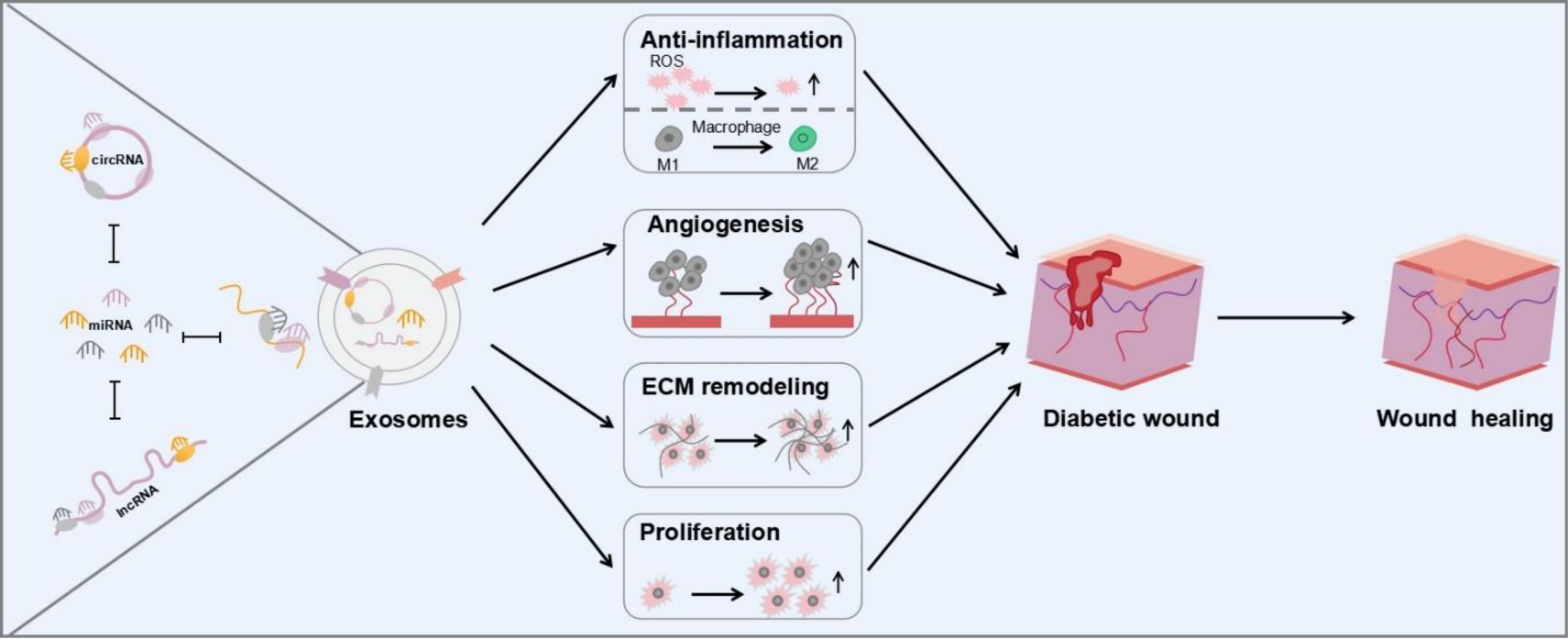
Schematic illustration of ncRNA carried by exosomes for diabetic wound healing.

Exosomes are extracellular vesicles with a diameter of about 30–150 nm, which serve as a vehicle to carry various substances, including various proteins, complexes, and non-coding RNAs. Exosomes play key roles in intercellular communication and the exchange of substances between cells. Exosomes contain a large amount of RNA species, including mRNA, miRNA, rRNA, lncRNA, tRNA, piRNA, snRNA, and snoRNA, which are involved in a variety of physiological and pathological processes (6, 7). Non-coding RNAs (ncRNAs) such as miRNA, lncRNA, and circRNA in exosomes are considered as active ingredients. ncRNAs are able to transcribe from the genome and perform their biological functions at the RNA level to regulate a variety of types of biological process. Particularly, ncRNAs delivered by exosomes may affect the occurrence and progression of various diseases *via* diverse mechanisms, such as diabetes. Some miRNA-containing exosomes regulated insulin sensitivity through targeting important protein key modulators and serving as pathological factors (8). There are strong associations between lncRNAs including ANARIL, MEG2, Sox2OT, MALAT1, and diabetes, especially type 2 diabetes (9). Additionally, circRNA and piRNA are known to promote the pathological process of diabetes (10). Abnormal expression of exosomal ncRNAs is observed in the wound of diabetic patients accompanied with elevated level of reactive oxygen species (ROS) and M1 macrophage polarization caused by high concentration of blood glucose. Therefore, these abnormal exosomal ncRNAs in exosomes could facilitate further the detection and treatment of diabetes. Garcia et al. have shown seven miRNAs with abnormal expression, which are isolated from plasma exosomes of diabetic patients. These miRNAs could be considered for the diagnosis of T1DM. On the contrary, some exosomal ncRNAs could be employed to treat diabetes and its complications by regulating several signaling pathways through regulating macrophage polarization toward M2 (11), accelerating fibroblast migration and proliferation, and promoting neovascularization (12) and angiogenesis (13). In addition, stem-cell-derived exosomes are well known to regulate inflammation and promote re-vascularization, re-epithelialization, and extracellular matrix remodeling in the repair of diabetic wounds *via* ncRNA. Besides miRNAs, lncRNAs and circRNAs, tRFs, which are tRNA-derived fragments, could regulate inflammatory response *via* the nuclear factor kappa B (NF-κB) signaling pathway. Liu and his colleagues have demonstrated that the nuclear translocation of p65 could be suppressed by decreasing the level of tRF5-AlaCGC, inhibiting NF-κB activity to reduce inflammation (14). More types of ncRNAs to accelerate the healing process will be discovered in the near future. In this review, ncRNAs in exosomes in terms of diabetic wound healing *via* anti-inflammatory, angiogenic, cell proliferation promoting, and matrix remodeling were reviewed (**Scheme 1**).

## Exosomes

Exosomes are first discovered in 1983 in the supernatant of sheep erythrocytes cultured *in vitro* as vesicles within the lumen of the cell. Exosomes have a density of 1.10–1.18 g/ml; are flat, spherical, or cup shaped under electron microscopy; and are mainly spherical in structure in body fluids (15). Exosomes are the end-products of the endocytosis process, in which cells were cytosol zed to form endosomal vesicles (ILVs) with exogenous antibodies, which formed early endosomes (EEs) by the action of organelles such as the Golgi apparatus. The vesicle membrane of EEs is constantly invaginated and selectively receives intracellular LEs fused with the cell membrane and releases exosomes into the extracellular environment (16, 17). In addition, exosomes demonstrate property to fuse with the cytosolic membrane of neighboring cells and then release the nucleic acid molecules carried by exosomes, such as DNA, mRNAs, miRNAs, and lncRNAs. In the meanwhile, exosomes bind to receptors to involve signal transduction and alter cellular function. They can also regulate cell status directly by transporting proteins and lipids, causing a series of biological effects in the target cells. In diabetic wounds, the expression levels of inflammation-related genes including p53, HIF-1α, TNF-α, IL-6, IL-10, NF-κB, and STAT1 are abnormal, leading to the slowing of wound healing (18, 19).

### Exosomal miRNA for diabetic wound healing

miRNAs are a class of small ncRNAs consisting of about 22 nucleotides that are widely found in plants, animals, and some viruses. They are involved in regulating physiological and pathological processes in the body by binding to the untranslated regions (UTRs) of target mRNAs and playing a role in transcriptional or post-transcriptional regulation (20); miRNAs can be encapsulated in exosomes, which carry and release them into target cells or tissues (21). In diabetic wounds, they are involved in regulating multiple fields in inflammatory response, angiogenesis and homeostasis, ECM generation, and re-epithelialization. Exosomal miRNAs are emerging key regulators of diabetes development and progression and have been demonstrated to play a vital role in regulating the progression of diabetic wound healing.

### Exosomal miRNAs regulated the inflammatory response

**Figure 2:**
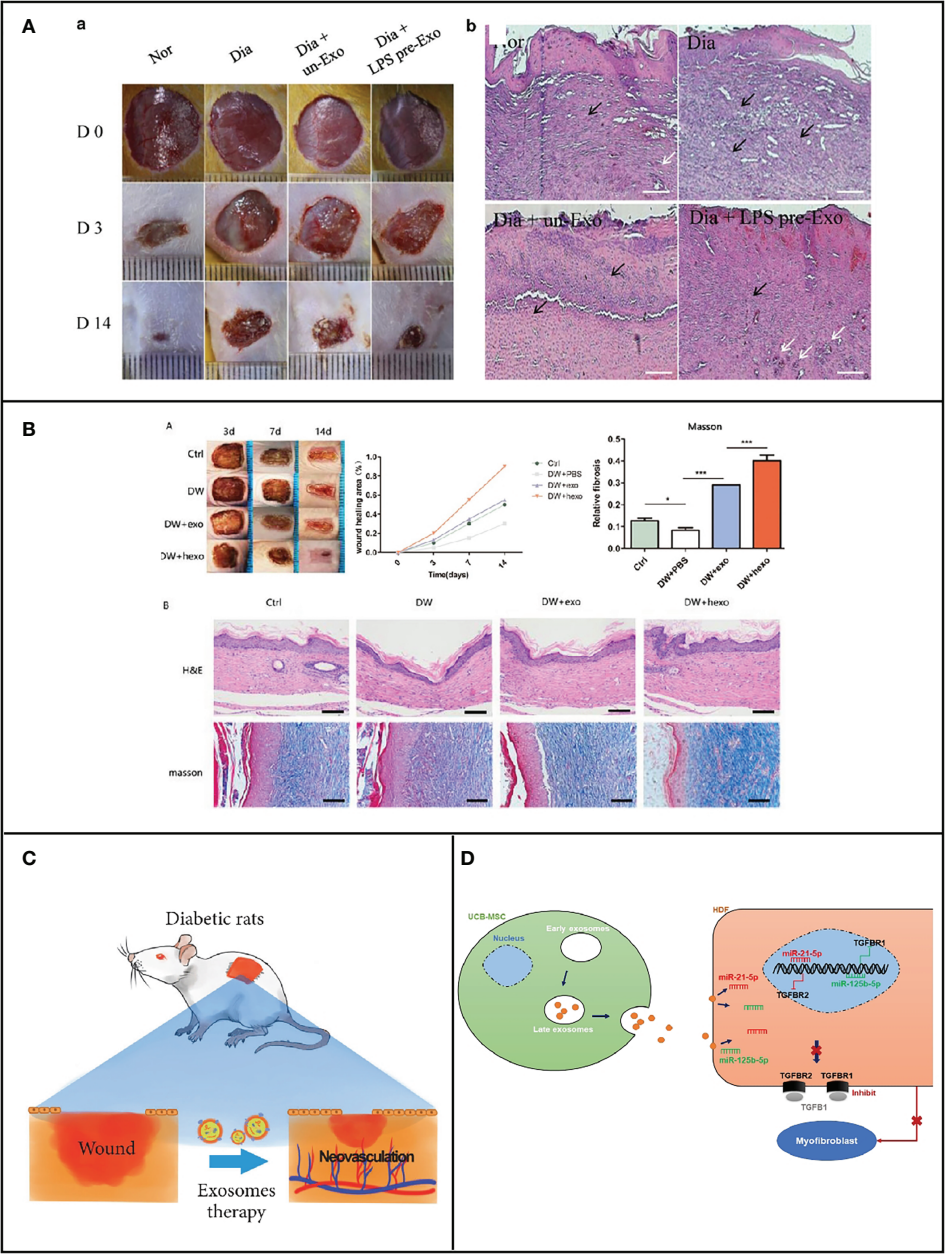
**(A)** miRNA carried by MSC-generated exosomes regulates macrophage polarization for the healing of cutaneous wounds in diabetic rats [Adapted from Ti et al., doi: 10.1186/s12967-015-0642-6, (28)]. **(B)** miRNA carried by ADSCs exosomes for diabetic wound healing [Adapted from Wang et al., doi: 10.1186/s12951-021-00942-0, (29)]. **(C)** Exosomes derived from marrow mesenchymal stem cells to accelerate cutaneous wound healing by promoting angiogenesis [Adapted from Ding et al., doi: 10.1155/2019/9742765, (30)]. **(D)** UCB-MSC-exo improves regenerative wound healing and suppresses scar formation by inhibiting the expression of TGF-β receptors [Adapted from Fang et al., doi: 10.5966/sctm.2015-0367, (31)].

In the process of diabetic wound healing, diabetic wound may stall at inflammation phase; the microenvironment for healing is thus disrupted, making wound healing poor. Monocytes and macrophages are widely considered to be responsible for the early inflammatory response to wounds, but they also facilitate in angiogenesis, wound contraction, and tissue remodeling. Macrophages are divided into two main subpopulations according to activities. M1 types promote inflammation and inhibit cell proliferation, while M2 types inhibit inflammation and promote cell proliferation. The transition from M1 to M2 type of wound macrophages to regulate the inflammatory response is the key factor in promoting wound healing. However, in diabetic wounds, there is an excessive impairment from M1 to M2 types, which resulted in these wounds not going through a normal healing state but always in a permanent inflammatory state (22). Mesenchymal stem cells (MSCs) are employed to treat various inflammatory responses in the wound healing process *via* the transformation of macrophage from M1 to M2. Currently, mesenchymal stem cell therapy (MSCT), a cell therapy, is considered as an attractively therapeutic method to enhance skin wound healing. During cutaneous wound healing, most of the therapeutic benefits of MSCT appear to because of paracrine signaling pathways through the stimulation of not only differentiation but also angiogenesis (23–25). However, the mechanisms by which bone marrow MSCs regulate inflammation remained unclear until 2019. He et al. found that inhibiting exosomes secretion that derive from MSCs resulted in the reduction in M2 macrophages in both *in vitro* co-culture systems and *in vivo* interaction sites. To sum up, MSCT is capable of inducing macrophage polarization to the M2 type by secreting exosomes. Further studies revealed that miRNA-223 in MSCT-EXO could modulate the polarization of macrophages to M2 by targeting the PHNOX1 gene, thereby regulating the inflammatory response in the wound and achieving a wound healing effect (11). Osteoarthritis (OA) pathology could be controlled by overexpression of miRNA because miRNA-210 can reduce the expression of proinflammatory cytokines (26). High-throughput sequencing results have revealed that the expression level of miRNA-210 is low in bone marrow mesenchymal stem-cell-derived exosomes (BMSCs-Exos). Hence, BMSCs-210-Exos-derived exosomes with overexpressing miRNA-210 could effectively inhibit chondrocyte apoptosis (27). In addition, MSCs can also be preconditioned with lipopolysaccharide (LPS), and treated MSCs show enhanced paracrine effects including increased nutritional support and improved reproductive and reparative properties. Ti et al. discovered that the exosomes secreted by MSCs that were pretreated with LPS have 40 significantly abnormal expressions of miRNAs, as compared to those without treatment (28). During these miRNAs, miR-let-7b have the highest expression levels of five unique miRNAs in hLPSpre-Exo. miR-let-7b in LPSpre-Exo could convert the polarization of macrophage *via* TLR4/NF-κB/STAT3/AKT signaling pathways. As a result, the inflammatory response was significantly attenuated, resulting in the promotion of diabetic wound healing (**Figure 2A**). Thus, miRNAs in exosomes could inhibit wound inflammation by modulating macrophage status, thereafter promoting wound healing.

### Exosomal miRNAs for fibroblast/keratinocytes proliferation

In chronic non-healing wounds, an abnormal phenotype including reduced proliferation, early senescence, and altered cytokine release patterns is observed in fibroblasts and keratinocytes (32). Fibroblasts have been found to promote granulation tissue formation by secreting extracellular matrix, and microfilaments and microtubules within fibroblasts can form a skeleton that acts as a fibrous scaffolding structure for wound repair (33). A loose connective tissue contains a large number of fibroblasts that produce elastic, collagenous, and reticular fibers, which play an essential role in wound healing. Therefore, the proliferation and migration of fibroblasts through exosomal miRNAs are effective tactics to accelerate wound healing. The proliferation of cells is enhanced after transplantation of stem cells. The generated stem cell exosomes show a similar function with stem cells to improve cell proliferation.

Fibroblast proliferation and migration could be improved when subjected to hypoxic adipose stem-cell-derived exosomes. Under hypoxic condition, the activity and proliferation of adipose stem cells are significantly enhanced compared to normoxia, which was reported by Wang and his colleagues (29). The expression of 215 microRNAs (miRNAs) was increased, in which 369 miRNAs were decreased in hypoxia adipose stem cell exosomes (HypADSCs-exo) in comparison with adipose stem cell exosomes (ADSCs-exo) on the basis of high-throughput sequencing. An increase in miR-21-3p, miR-126-5p, and miR-31-5p and a decrease in miR-99b and miR-146-a were observed. Furthermore, the inflammation was inhibited after treatment with the above-mentioned exosomes *via* PI3K/AKT signaling pathway. Meanwhile, cell metabolism, differentiation, and TGF-β function were also regulated. As can be seen in **Figure 2B**, the process of diabetic wound healing was accelerated when subjected to HypADSCs-exo. Therefore, stem cell exosomes are an alternative approach in the promotion of diabetic wound healing. Besides ADSCs-exo, human amniotic mesenchymal stem cell (hAMSC)-derived exosomes could strengthen the migrating ability of fibroblast. Importantly, Chen et al. have demonstrated that the ability to improve wound healing in exosomes secreted by hAMSC was higher than that of unmodified hAMSC, while the above-mentioned ability of hAMSC-Exo was reduced after knockdown of miR-135a, suggesting that miR-135a in hAMSC-Exo can promote fibroblast proliferation and migration (34), which further indicated that exosomal miRNA is capable of helping wound shut. Moreover, miRNA also could be loaded into exosomes to corresponding function. Chen et al. indicated that these stem cell exosomes could promote the closeup of rat dorsal wounds. First, miRNA-146a was loaded into adipose stem cell exosomes. Subsequently, wound healing was improved because those exosomes could enhance the migration and proliferation of fibroblasts and accelerate neovascularization through the regulation of the level of SERPINH1 and p-ERK (35). Exosome cargo miRNA-146a was demonstrated to inhibit the leukocyte adhesion molecules expression and the level of proinflammatory cytokines, causing suppressive effect toward the inflammation of endothelial cells, which was activated by IL-1β. miRNA in exosomes could play multiple roles including the promotion of cell proliferation, migration, tube formation, and angiogenesis and the suppression of inflammatory behavior to the contribution of diabetic wound healing (12).

### Exosomal miRNAs for vascular growth

Chronic wounds caused by diabetes often have microangiopathy, accompanied with alterations such as decreasing production of pro-angiogenic factors and lessened endothelial progenitor cells, ultimately leading to abnormal function of the vascular network (36). Revascularization and angiogenesis are important manifestations of wound healing. miRNA-221-3p could improve the function of cells through regulating HIF-1α. Macrophages are polarized into proinflammatory by inhibiting JAK3 signal pathway (37). Xu et al. have reported the high expression of miRNA-221-3p in exosomes generated from endothelial progenitor cell (EPCs). Exosomal miRNA-221-3p can downregulate the expression of p27 and p57 proteins, which are involved in the cell cycle signaling pathway. According to immunohistochemistry results, the angiogenesis-related proteins VEGF and CD31 and the cell proliferation marker Ki67 had higher expression levels after treatment with EPC-derived exosomes with high miRNA-221-3p expression. Moreover, it has been revealed that miRNA-221-3p may be engaged in the cell cycle, AGE-RAGE, and p53 signaling pathway in diabetic complications. Therefore, miRNA-221-3p may encourage the proliferation of vascular cells by blocking cell cycle negative regulators (38). Additionally, miRNA-221-3p in stem cell exosomes activated Akt/endothelial nitric oxide synthase pathway to promote angiogenesis. Subsequently, the proliferation and migration of endothelial cell was enhanced, resulting in the improvement of diabetic wound healing (39). Other signaling pathway such as HIPK2 was targeted by exosomal miRNA-221-3p to improve the function of HUVEC and accelerate angiogenesis, contributing to concrescence of the wound in diabetic rats (40). On the other hand, Ding et al. found a significant increase in miR-126 levels upon treatment with deferoxamine exosomes (DFO-Exos) from BMSCs pretreated with deferoxamine (**Figure 2C**). It was also observed that when DFO-Exos was given, the amount of the phosphatase and tensin homolog (PTEN) in endothelial cells, one target of miRNA, was decreased. PTEN was a proangiogenic pathway in endothelial cells *via* the PI3K/AKT signaling pathway. When miR-126 was inhibited, the level of PTEN was increased. Thus, miR-126 can promote angiogenesis by decreasing the level of PTEN (30). Similarly, Xiong et al. observed a distinct increase in miR-20b-5p in exosomes, which was obtained from plasma of patients with T2DM. Angiogenesis in human umbilical vein endothelial cell was inhibited by miR-20b-5p through Wnt9b/β signaling pathway. Either miR-20b-5p or diabetic exosomes, which was exposed to the wound site, was sufficient to slow down both wound healing and angiogenesis. Therefore, diabetic wound healing could be improved after treatment with mir-20b-5p inhibitors including ncRNA (41). Engineered exosomes with miRNA-21-5p from human adipocytes accelerate wound healing in a model of full-thickness skin defects in diabetic rats; the results of diabetic rats and keratinocytes indicated that miRNA-21-5p functions through the Wnt/b-catenin pathway and promotes angiogenesis and collagen remodeling in the wound area (42). miRNA-21-5p in exosomes derived from human mesenchymal stem cells promotes the proliferation of HUVECs and enhances angiogenesis by activating Akt and mitogen-activated protein kinase (MAPK) and upregulating VEGFR1. Meanwhile, miRNA-21-5p accelerates the wound healing of foot ulcers in diabetic rats (43). In addition, Yan et al. have utilized low-cost milk exosomes, which served as vehicle to load miR-31-5p by electroporation. the exosomal miR-31-5p dramatically improved endothelial cell function, leading to enhanced diabetic wound healing by downregulating the expression of HIF1AN process (13).

### Exosomal miRNAs for matrix remodeling

The body avoided pathogen infection and rehydrated in the presence of epidermal cells. The extracellular matrix is mainly composed of collagen, fibronectin, elastin, laminin, etc. It provides an appropriate microenvironment for cells, thus inducing cell adhesion, migration, and differentiation (44). During wound repair process, the extracellular matrix is an essential constituent. The related components secreted by keratinocytes are indispensable parts for the skin to function normally (45). In the early stages of healing, collagen deposition is more significant. Fibroblast could produce large quantities of collagen and other matrix components to construct the microenvironment for epidermal cell coverage (46). However, in the later period of healing, matrix remodeling is more important. The remodeling of the extracellular matrix usually takes 2 weeks or even 1 year, and the production and remodeling of the extracellular matrix are critical in the extent of scar formation. Because of similar functions with stem cells, stem cell-derived exosomes could be employed to regulate the extracellular matrix and promote collagen production, thereby reducing scarring. As demonstrated in **Figure 2D**, Fang et al. indicated that umbilical-cord-derived bone marrow mesenchymal stem cells (uMSCs) reduced scar formation and myofibroblast accumulation in a mouse model of skin defects. By utilizing miRNA omics, the uMSC-Exos group, enriched in specific miRNAs (miR-21, miR-23a, miR-125b, and miR-145), played a key role in inhibiting myofibroblast formation through suppressing the TGF-β/SMAD2 pathway (31). Exosomes secreted by human amniotic fluid stem cells also prevent myofibroblast differentiation. Zhang et al. assessed the impact of hAFSC-derived exosomes (hAFSC-exo) on anti-fibrotic scarring during wound healing by employing a rat model of complete skin damage. Through exosome-specific miRNAs, hAFSCs aid in the healing of wounds and inhibit the development of fibrotic scars. Additionally, hAFSC-exo-specific miRNAs enhance scar-free wound healing by the downregulation of TGF-R1 and miR-21-5p, miR-22-3p, and miR-27a-3p and the inhibition of myofibroblast production (47).

### Exosomal lncRNAs regulated the inflammatory response

**Figure 3:**
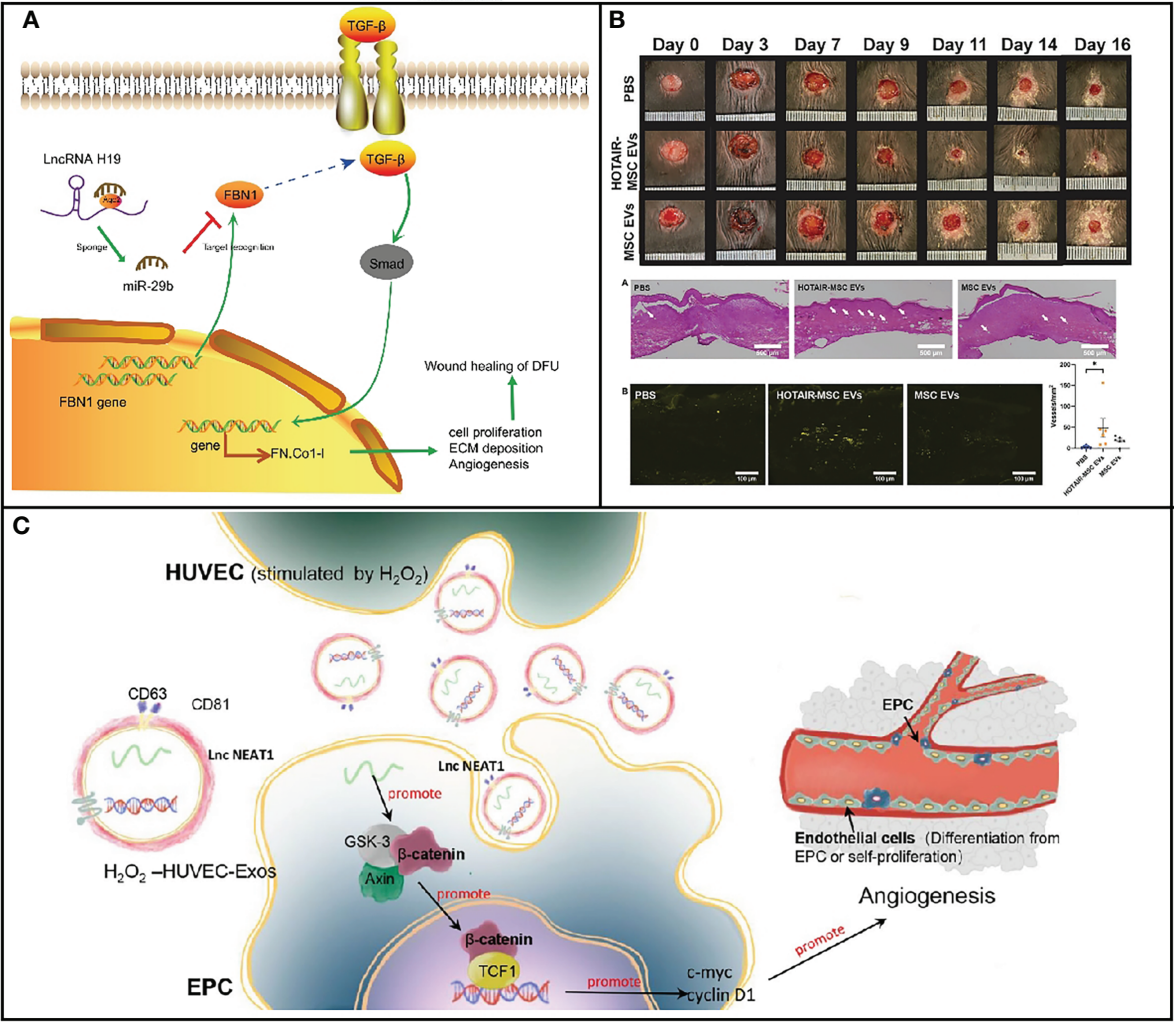
**(A)** Map of molecular mechanisms involved in lncRNA H19 modulation in wound healing of DFU by binding to miR-29b to regulate FBN1 expression [Adapted from Li et al., doi: 10.1096/fj.201900076RRRRR, (49)]. **(B)** HOTAIR-MSC EVs improve wound healing in db/db mice *via* increasing angiogenesis [Adapted from Born et al., doi: 10.1002/adhm.202002070, (50)]. **(C)** Schematic illustration of the function and mechanisms of HUVEC-Exos on EPCs [Adapted from Guo et al., doi: 10.1186/s13287-022-03013-9, (51)]. *p < 0.05.

For diabetes, lncRNA plays significant roles in regulating wound inflammation *via* several pathways. ROS overproduction triggers neutrophil inflammatory infiltration, increased protease release, and an abundance of oxidative intermediate products, which ultimately result in cell death, affecting the healing up of wound in diabetic patients (48). The activity of transcription factors is directly regulated by low amounts of ROS as a signal molecule, which reduces apoptosis. Cells subjected to H_2_O_2_ showed a strong capacity to promote tube formation, migration, and proliferation of EPCs in a dose-dependent manner. In diabetic wounds, the expression level of inflammation-related genes including p53 was abnormal, leading to slow wound healing. PI3K/AKT was reported to act as a protective role against apoptosis caused by high glucose. PTENs have been demonstrated as a target gene of miRNA-152-3p, and lncRNA-H19 was a transcript antisense RNA affecting the level of miRNA-152-3p. PTEN serves as antagonist of PI3K. In 2019, Li and his colleagues found a high expression of miRNA-152-3p and low expression of lncRNA-H19 in the fibroblast with diabetic foot ulcer. After treatment with lncRNA-H19-overexpressed MSC-derived exosomes, miRNA-152-3p decreased in fibroblast accompanied by not only apoptosis but also inflammation (**Figure 3A**). In addition, proliferation and migration in fibroblast were promoted. As a consequence, the process of wound healing was accelerated upon injection with overexpressed lncRNA-H19 exosomes (52). Upregulation of lncRNA-H19 can also elevate the expression of FBN1 through competitively binding to miR-29b, which enhances the proliferation, migration, and inhibits apoptosis of fibroblasts, thus facilitating the wound healing of DFU (49). Exosomes that are secreted by the above-mentioned cells also have similar functions to cells. Furthermore, lncRNA NEAT1 in exosomes shows overexpression stimulated by oxidative stress on EPC cells, which is the effective therapeutic ingredient of exosomes (**Figure 3C**) (51). Evidence suggested that ROS concentration in macrophages could be modulated by lncRNA Lethe and lncRNA NTF3-5. The biochemical mechanism is that lncRNA NTF3-5 suppresses the level of NOX2 gene by activating NF-κB signaling on account of negative feedback loop between lncRNA Lethe and NF-κB (53). Similarly, increasing the expression of lncRNA MALAT1 is an alternative strategy to promote wound healing in diabetic patients (9). These lncRNAs, which could be cargoes by exosomes *via* various methods, would be employed as therapeutic drugs for the acceleration of the process of wound healing.

## Exosomal lncRNA for diabetic wound healing

The lncRNA is a loosely classified group of long RNA transcripts with no apparent protein-coding role with a length >200 nucleotides. LncRNAs play a role in several significant biological processes, including the imprinting of genomic loci, sculpting of chromosome shape, and allosteric control of enzyme function. LncRNA performs the functions through regulating target genes with different mechanisms such as scaffolds, decoys, signals, and guide. Thus, lncRNAs are associated with cell differentiation, status, development, and illness. Among these lncRNAs, X-inactive-specific transcript (XIST; in X chromosome inactivation), HOX transcript antisense RNA (HOTAIR; in positional identity), and telomerase RNA component (TERC; in telomere elongation) were well-explored lncRNAs in functional roles and mechanisms.

### Exosomal lncRNAs for fibroblast/keratinocytes proliferation

He and coworkers demonstrated that the content of lncRNA CASC2 in the tissue was reduced in diabetic foot ulcers. The wound healed speedily when the lncRNA CASC2 was overexpressed. LncRNA CASC2 abolished fibroblasts apoptosis and promoted cell proliferation and migration. Mechanically, lncRNA CASC2 interacted with miRNA 155 to upregulate the level of its target gene HIF-1α. Thus, overexpression of lncRNA CASC2 is a novel approach for diabetic wound healing. Increasing HIF-1α level by lncRNAs is considered as an effective strategy to improve healing process. LncRNA-H19 and lncRNA MIR31HG have been employed to regulate the expression of HIF-1α to enhance the proliferation of fibroblast (54). In addition, lncRNA FAM83A-AS1 involved several signaling pathways such as glycolysis, hypoxia, and OXPHOS signaling. Chen et al. proved that lncRNA FAM83A-AS1 could enhance cell migration, invasion, and proliferation (55). LncRNA FAM83A-AS1 may be applied to strengthen the proliferation of fibroblast/keratinocytes for the treatment of diabetic wound. Although there is no literature on exosomal lncRNAs for cell proliferation in wound healing, lncRNAs delivered by exosomes would be explored in the near future.

### Exosomal lncRNAs for vascular growth

LncRNA in exosomes is capable of promoting angiogenesis to accelerate wound healing. HOTAIR has been shown to promote angiogenesis in several setting. Jay’s group has constructed exosomes with increased HOTAIR content to enhance angiogenesis for the promotion of healing of chronic wounds. On account of its intrinsic angiogenic properties, exosomes that were isolated from MSC with overexpressed HOTAIR demonstrated high level of HOTAIR compared with MSC without treatment. After treatment with HOTAIT-MSC exosomes, HOTAIR content was as much as 21-fold in human umbilical vein endothelial cells (HUVECs), resulting in about 11-fold increase in VEGFA within HUVECs. Moreover, gap closure was distinctly improved when exposed to HOTAIR-MSC exosomes (**Figure 3B**). As a consequence, there was an increase in the number of vessels in healed tissue when subjected to HOTAIR-MSC exosomes, demonstrating that lncRNA delivered by exosomes could be employed as nanomedicine for wound healing. Therefore, stimulation of angiogenesis and VEGFA are general therapeutic strategies to promote wound healing through lncRNA carried by exosomes (50). Similarly, Han et al. constructed MSC-derived exosomes to deliver lncRNA KLF3-AS1 to accelerate wound healing by the upregulation of VEGFA. KLF3-AS1 acted as a competing endogenous RNA for MiR-383 to increase the VEGFA signaling for diabetic wound healing. Under diabetic condition, exosomal KLF3-AS1 significantly promoted the proliferation of HUVESs. The content of proapoptotic proteins markedly upregulated, while anti-apoptotic proteins reduced after treatment with lncRNA delivered by exosomes due to the increasing VEGFA expression level. Interestingly, inflammation was also suppressed in the KLF3-AS1-Exos group, which further improved curative effects. Thus, improving angiogenesis and suppressing inflammation are effective strategies to treat diabetic wound (56).

### Exosomal lncRNAs for matrix remodeling

A research by Herter at al. demonstrated that the expression of WAKMAR2 (lncRNA) was decreased in human chronic wounds as compared to normal wounds (57). WAKMAR2 is an RNA polymerase, which is observed in the cytoplasm and in the nucleus of keratinocytes. In order to investigate the molecular mechanisms modulation, the level of WAKMAR2, a variety of biomolecules, was used to treat keratinocytes in the wound, indicating that WAKMAR2 was regulated by TGF-β signaling pathways. Furthermore, the migration of keratinocytes was enhanced, and inflammation was suppressed in the presence of WAKMAR. As a consequence, diabetic wound repair could be improved when subjected to WAKMAR2, especially WAKMAR2 delivered by exosomes. Proteins in ECM could be degraded and modified by enzymes such as MMPs, serine proteinases, and disintegrant (58). Ye’s group indicated that cancer cell proliferation and migration were accelerated by lncRNA P73 antisense RNA 1T (lncRNA TP73-AS1) through regulating the expression of MMP2 and MMP9. By utilizing lncRNA TP73-AS1 carried by exosomes, the healing process may be accelerated *via* downregulating the proteins level of MMP2 and MMP9 to decrease degradation of ECM proteins.

## Exosomal circRNA for diabetic wound healing

CircRNAs are a class of coding/ncRNA molecules that form a closed loop of exons and/or introns by covalently binding at the 3′and 5′ends (59, 60). Compared to linear mRNAs, circRNAs contain more abundant transcripts and are capable of regulating gene expression at the transcriptional and post-transcriptional levels. circRNAs are components of competing ceRNAs that could regulate gene expression by inhibiting miRNA activity to perform functions in physiological processes such as cell cycle or aging (61–63). They have tissue cell selectivity, structural stability, and sequence conservation and are widely expressed in mammalian cells.

### Exosomal lncRNAs regulated the inflammatory response

Circ-Snhg11 from hypoxia-pretreated ADSC exosome promoted diabetic wound healing by increasing vascular differentiation function of EPCs by activation of the miR-144–3p/HIF-1α/VEGF signaling pathway. The result also found that circ-Snhg11 can also promote M2-like macrophage polarization under HG conditions by the regulation of miR-144–3p/HIF-1α/STAT3 signaling pathway (64). A research by Wang at al. discovered that the decrease in circRNA 001372 could upregulate IL-1, IL-6, IL-17, and IL-18, resulting in damage and apoptosis of neurocyte (65), while the inflammatory response was reduced when circRNA 001372 was overexpressed. Furthermore, they revealed that circRNA 001372 targeted miRNA-148b-3p to suppresses inflammation *via* the PI3K/Akt/NF-κB signaling pathway.

### Exosomal circRNAs for fibroblast/keratinocytes proliferation

Si et al. have found that circRNA_100797 expression was decreased in human fibroblast after exposure to ultraviolet B. miR-23a-5p was the target of circRNA_100797, as analyzed by Gene Ontology and quantitative real-time PCR (qRT-PCR). The proliferation of fibroblast was enhanced when circRNA_100797 was highly expressed, and the cell cycle arrest was alleviated (66). In addition, the level of circRNA_000203 in cardiac fibroblast in diabetic mouse was increased accompanied by the increase in Col1a2, Col3a1, and α-SMA (67). circRNA_000203 could especially bind to miR-26b-5p for the suppression of the interaction between miR-26b-5p and collagen. Although circRNA_000203 exerted negative function in cardiac fibroblast, it could promote the healing of wound on account of promoting the expression of collagen. Therefore, circRNA serves as a sponge of miRNA to improve fibroblast/keratinocytes proliferation for the promotion of diabetic wound healing, especially delivered by exosomes by taking advantage of its perfect biocompatibility.

### Exosomal circRNAs for vascular growth

**Figure 4:**
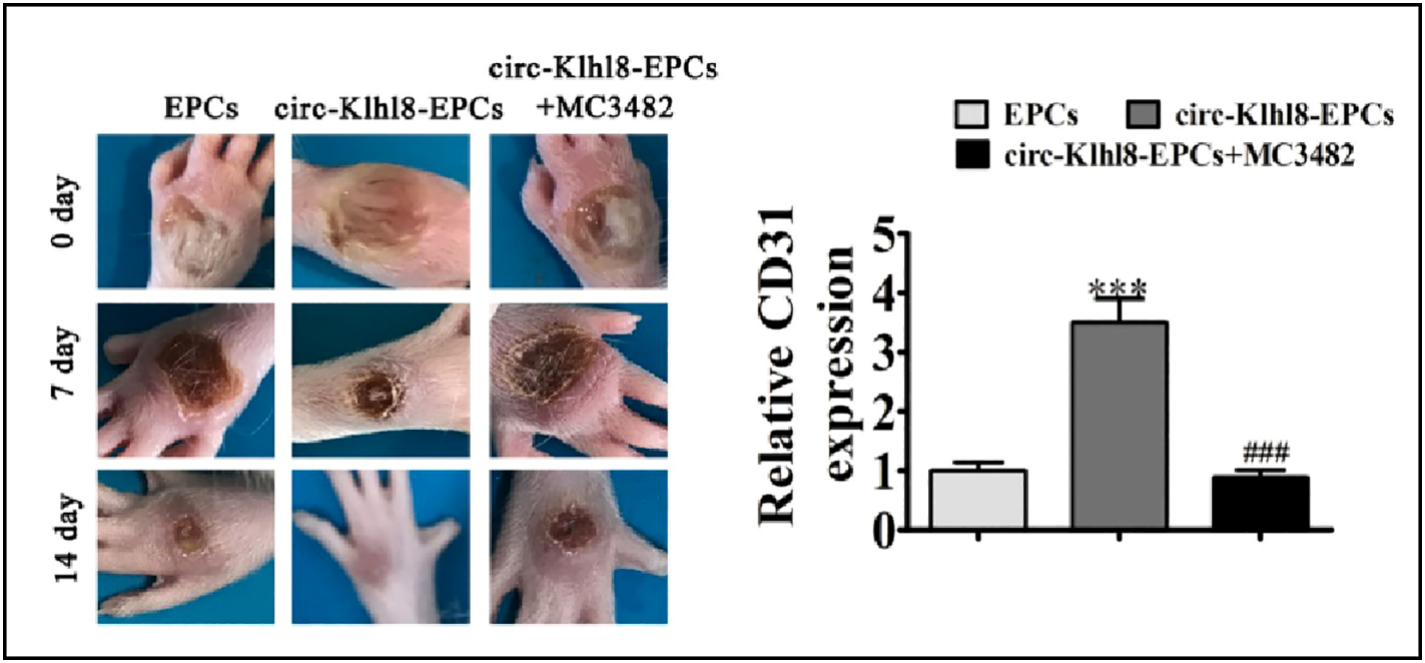
Hypoxic pretreatment endothelial progenitor cell (EPCs) accelerates diabetic wound closure and promotes angiogenic [Adapted from Shang et al., doi: 10.1016/j.jdiacomp.2021.108020, (71)]. ***p < 0.001 vs EPCs. ^###^P < 0.001 vs circ-Klhl8-EPCs.

It has been noted that hyperglycemia (HG) levels can affect the amounts of circRNA expression in HUVECs. Three of these circRNAs (hsa circ 0008360, hsa circ 0000109, and hsa circ 0002317) showed the largest upregulation, according to the miRNA genomics, which identified 214 circRNAs with differential expression. Based on bioinformatics, these circRNAs control the expression of genes involved in angiogenesis and vascular endothelial function (68). Chen et al. compared the differences in exosomal circRNAs isolated from serum between diabetic and non-diabetic patients. There were 67 circRNA differences in the level of expression between the two groups, and in the DFU group, 28 circular RNAs were upregulated and 39 were downregulated (69), circRNA not only could be used as serum biomarker for the clinical diagnosis of DFU, but it also plays a role in diabetic wound healing. Angiogenesis is an important cause of diabetic wound healing. One potential cause of diabetic ulcers is the damage to vascular endothelial cells, and EPC transplantation is considered as a promising approach for the treatment of hypovascular chronic wounds (70). In their study, Shang et al. revealed that transplantation of hypoxia-pretreated pre-endothelial progenitor cells (EPCs), which were under hypoxic condition before use, was more therapeutically effective than EPCs without treatment in accelerating diabetic wound healing. The overexpression of circ-Klhl8 inhibited hyperglycemia, which caused the injury to endothelial cells *via* activating autophagy. miR-212-3p was verified as the sole target of circKlhl8 by employing bioinformatics. miR-212-3p in diabetes causes pancreatic β-cell dysfunction and inhibits autophagy (71). In contrast, a certain level of autophagy promotes EPC migration and angiogenesis, and EPCs overexpressing circ-Klhl8 can promote wound repair by sponging miR-212-3p to reduce its expression and activate autophagy (**Figure 4A**). circ-Klhl8 can also regulate autophagy to accelerate wound healing by promoting SIRT5 overexpression. Additionally, it has been demonstrated that ADSC-exo carrying mmu circ 0000250 stimulates the production of miR-128-downstream 3p’s gene SIRT1 by binding to it and then activates cellular autophagy. Exosomes with high concentrations of mmu circ 0000250 sped up the healing of diabetic wounds (**Figure 4B**). It has been demonstrated that exosomes could increase angiogenesis and autophagy in Exosomes derived from human umbilical cord blood mesenchymal stem cells stimulate skin wounds to accelerate its healing (72).

## Challenge and perspective

At present, most of the research on ncRNA in diabetic wound repair is based on the role of naked nucleic acid. However, due to various shortcomings such as poor stability and deficiency of poor targeting, ncRNA have limited efficacy in clinical application and cannot be used for further applications. Conversely, exosomes containing nucleic acid can remain in the body for a longer time and have a stronger effect than naked nucleic acid drugs. However, the crucial problem is that rare sources of these natural exosomes cannot meet clinical needs. In addition, the content of ncRNA in exosomes does not suffice for clinical application. Therefore, ncRNA in engineered exosomes serves as cargo, which is loaded into exosomes. By taking advantage of biological, physical, chemical, and other methods, ncRNA could be packaged into exosomes with the same pharmacokinetic behavior with exosomes. However, the low envelopment rate is achieved by utilizing these passive loading methods. Active loading method will improve the envelopment rate for exosomes to load ncRNA. In addition, most of above approaches are inapplicable for large-scale production due to low yield and high cost. To address the above problems, biomimetic exosomes that possess similar structures with exosomes and liposomes can also be used as a substitute for natural exosomes to delivery ncRNA. Generally, biomimetic exosomes could be prepared *via* top–down and down–top strategy by employing lipids, proteins, and RNA or cells. Biomimetic exosome drugs have the characteristics of clear source, stable physicochemical properties, low cost, and good biocompatibility as compared to liposomes. Furthermore, compared with natural exosomes, biomimetic exosomes are easier to be synthesized on a large scale in comparison with natural exosomes isolated from cells. Nevertheless, clinical trials of engineered exosomes have just begun, and the synthetically biomimetic exosome have not yet been approved for further clinical application on account of lack of standard approaches for production, characterization, and quality control. In addition, some of the ncRNAs could involve several signaling pathway with multiple targets, which is not beneficial as a drug. In the future, ncRNA carried by novel multifunctional engineered exosomes and biomimetic exosomes will be generated by biotechnology, providing a new avenue for diabetic wound therapy.

## Conclusion

Due to the significant impact on the quality of life of complications induced by the diabetes especially diabetic trauma, diabetic wound healing is urgently needed to improve patients’ life quality. Diabetic wound healing is affected by inflammation, cell proliferation, angiogenesis, and ECM remodeling. ncRNA could regulate these conditions to promote diabetic wound healing (**Table 1**). In comparison with synthetic vehicles, exosomes as a vehicle are employed to deliver ncRNA owing to high biocompatibility and lower toxicity. The level of specific ncRNA in natural exosomes does not fulfill clinical application such as diabetic wound healing. To date, a few exosomes for diabetic wound healing is undergoing clincal trials (**Table 2**). Therefore, engineered exosomes are constructed by various strategies including biological, physical, chemical, and other methods to carry ncRNA. Although several challenges need to be overcome in terms of ncRNA delivered by exosomes for clinical applications, there will is a broad prospect for this medicine in wound healing.

**Table 1:** Molecular mechanisms and therapeutic potential of exosomal ncRNAs involved in diabetic wound healing.

Exo-ncRNAs	Molecular mechanisms	Final effect	Ref.
miR-223	Regulate macrophage polarization by targeting pknox1	Reduce inflammatory response	(11)
miR-let-7b	convert the polarization of macrophage *via* TLR4/NF-κB/STAT3/AKT signaling pathways	Reduce inflammatory response	(28)
miR-135a	Inhibit E-cadherin, N-cadherin, and LATS2 and promote α-SMA expression	Promote fibroblasts migration	(33)
miR-146a	Upregulate SERPIN and p-ERK2 expression in fibroblasts	Promote fibroblasts migration	(29)
miR-221-3p	Increase the expression of VEGF, CD31, and Ki67 involved in AGE-RAGE and p53 signaling pathway, cell cycle	Promote vascular cells proliferation	(38)
miR-126	activate the PI3K/AKT signaling pathway *via* miR-126-mediated PTEN downregulation	Promote angiogenesis	(30)
miR-21、-23a、-125b、-145	Inhibit TGF-b2, TGF-bR2, and SMAD2 and suppress α-SMA and collagen I expression	Anti-scarring effect	(31)
lncRNA-H19	Impair miR-152-3p-mediated PTEN inhibition	Inhibit apoptosis and inflammation, promote fibroblasts migration	(52)
lncRNA KLF3-AS1	Decrease miR-383 expression and upregulate VEGFA	Increase angiogenesis, reduced inflammation	(56)
lncRNA NEAT1	mediate Wnt/β-catenin signaling pathway	Increase angiogenesis	(51)
circKlhl8	Activate miR-212-3p/SIRT5 signaling pathway	Increase survival and maintain endothelial function in EPCs	(71)
mmu circ 0000250	Induce miR-128-3p/SIRT1 mediated autophagy	Increase angiogenesis and autophagy	(72)

**Table 2:** Summary of the exosome for diabetic wound healing in clinical trials.

NCT Number	Title	Status	Conditions	Interventions	Phases	Year
NCT05475418	Pilot Study of Human Adipose Tissue Derived Exosomes Promoting Wound Healing	Not yet recruiting	Wounds and Injuries	Procedure: Adipose tissue derived exosomes	Not Applicable	2022
NCT02565264	Effect of Plasma Derived Exosomes on Cutaneous Wound Healing	Unknown status	Ulcer	Other: plasma-derived exosomes	Early Phase 1	2015
NCT05243368	Evaluation of Personalized Nutritional Intervention on Wound Healing of Cutaneous Ulcers in Diabetics	Not yet recruiting	Foot, Diabetic	Dietary Supplement: Personalized Nutritional Intervention	Not Applicable	2021
NCT04849429	Intra-discal Injection of Platelet-rich Plasma (PRP) Enriched with Exosomes in Chronic Low Back Pain	Completed	Chronic Low Back Pain| Degenerative Disc Disease	Biological: Platelet rich plasma (PRP) with exosomes Drug: Normal Saline	Phase 1	2019
NCT04134676	Therapeutic Potential of Stem Cell Conditioned Medium on Chronic Ulcer Wounds	Completed	Chronic Ulcer	Drug: Conditioned Media	Phase 1	2018
NCT02768935	Macrophage Phenotype in Type 2 Diabetics After Myocardial Infarction and the Potential Role of miRNAs Secreted	Completed	Diabetes Myocardia Infarcts	Procedure: blood sample	Not Applicable	2017

## Author contributions

JS, XDW, XZ, and WA developed the review outline and drafted and wrote the manuscript. YZ and PY developed the figures. JS conceived the idea and supervised the content and writing. PG and XW critically contributed to the content and reviewed the manuscript to ensure accuracy and completeness. All authors contributed to the article and approved the submitted version.

## Conflict of interest

All authors were employed by China National Biotech Group.

## Publisher’s note

All claims expressed in this article are solely those of the authors and do not necessarily represent those of their affiliated organizations, or those of the publisher, the editors and the reviewers. Any product that may be evaluated in this article, or claim that may be made by its manufacturer, is not guaranteed or endorsed by the publisher.
